# Intra-articular injection of photo-activated platelet-rich plasma in patients with knee osteoarthritis: a double-blind, randomized controlled pilot study

**DOI:** 10.1186/s12891-016-0920-3

**Published:** 2016-02-09

**Authors:** Kade L. Paterson, Melissa Nicholls, Kim L. Bennell, Dan Bates

**Affiliations:** Centre for Health, Exercise & Sports Medicine, Department of Physiotherapy, Melbourne School of Health Sciences, The University of Melbourne, Melbourne, Victoria 3010 Australia; Lakeside Sports Medicine Centre, Melbourne, Victoria Australia; School of Exercise Science, Australian Catholic University, Melbourne, Victoria Australia

**Keywords:** Cartilage, Pain, Arthritis, Musculoskeletal diseases

## Abstract

**Background:**

Improvements in knee osteoarthritis (OA) symptoms with platelet-rich plasma (PRP) have been attributed to its ability to modify intra-articular inflammatory processes. Photo-activation of peripheral blood also improves inflammatory mediators associated with OA, however combined photo-activated PRP (PA-PRP) has not been investigated. This pilot study assessed the feasibility, safety and symptomatic and functional change following injections of PA-PRP compared to hyaluronic acid (HA) in people with knee osteoarthritis (OA).

**Methods:**

Thirty seven people with knee OA were enrolled in this double-blind randomized controlled pilot study set in a sports medicine clinic. Participants were randomly allocated to receive three injections of either PA-PRP or HA. The patients and the administering doctor were blinded to group allocation. Outcomes included recruitment and safety data, 100 mm visual analogue pain score (VAS), the Knee Osteoarthritis Outcome Score (KOOS), Knee Quality of Life (KQoL) scale, maximum hopping distance and number of knee bends in 30 s at four and 12 weeks.

**Results:**

Twenty three (62 %) participants met the inclusion criteria, of which 12 (32 %) were randomized to the PA-PRP group and 11 (30 %) to the HA group. Two participants did not complete the intervention and two withdrew following their first assessment. Minor pain and swelling during the injection period was reported by two participants from the PA-PRP group. The PA-PRP group demonstrated significant improvements in the VAS (*p* < 0.01, ETA = 0.686), KOOS Pain (p < 0.05, ETA = 0.624), KQoL Physical (*p* < 0.05, ETA = 0.706) and KQoL Emotional subscales (*p* < 0.05, ETA = 0.715) at four and 12 weeks. The PA-PRP group also significantly improved hoping (*p* < 0.05, ETA = 0.799) and knee bends (*p* < 0.01, ETA = 0.756) at four or 12 weeks. The HA group showed improvements on only the KOOS Function subscale at 12 weeks (*p <* 0.01, ETA = 0.602). After controlling for baseline values, there were no significant between-group differences at either time-point.

**Conclusions:**

This study provides proof-of-concept evidence concerning the feasibility and safety of PA-PRP injections necessary to inform a larger clinical trial in people with knee OA. Our preliminary results also suggest PA-PRP improves self-reported pain, symptoms and lower extremity function, however no between-group differences were found. Photo-activated PRP may provide a safe and effective novel treatment for knee OA.

**Trial registration:**

ACTRN12611000651987

## Background

Osteoarthritis is a leading cause of musculoskeletal pain worldwide and the knee is one of the most commonly affected joints. Prevalence of knee OA is expected to increase with an aging population and growing rates of obesity, and projections of total knee replacements are predicted to increase by approximately 600 % over the next 25 years [1]. As there is currently no cure for OA, treatment has focused on symptomatic relief with the aim of reducing pain and disability and maintaining or improving joint mobility [2]. Non-surgical treatments including exercise and weight loss are recommended due to poor symptomatic and functional outcomes with surgical management [3]. However compliance with non-surgical treatments is poor [4], whilst drug treatments such as simple analgesics and non-steroidal anti-inflammatory drugs are associated with adverse events [2, 5, 6]. The addition of intra-articular injections with hyaluronic acid (HA) products (viscosupplementation) has also been recommended in patients unresponsive to non-pharmacological or analgesic regimes [6], although this treatment is also uncertain as efficacy is variable and ongoing treatment is required [7–9]. Given the progressive nature of knee OA, and the serious limitations associated with existing therapies, studies in to effective treatments with potential disease-modifying effects are needed.

Recent research suggests that growth factors and other cytokines released by platelets in response to injury or pathology may modulate inflammatory processes and contribute to the maintenance or regeneration of tissue structures [10, 11]. Consequently, platelet-rich plasma (PRP) injections have become an emerging treatment for soft tissue healing associated with tendon and ligament injury, bone mineralisation and cartilage regeneration [12–14]. Upon application to the affected site, activated platelets release growth factors and other bioactive molecules, and coagulation occurs to form a matrix that promotes migration of additional cells to the area. Combined, these factors may promote tissue healing and modulate the aberrant inflammatory processes implicated in the pathophysiology of OA [10–12].

Recent unblinded and non-randomized pilot and prospective studies investigating the clinical efficacy of intra-articular injections of PRP in patients with knee OA have demonstrated clinical improvement in self-reported pain and symptoms with no major adverse events [10, 15–17]. Furthermore, a recent systematic review found six randomized controlled trials reporting clinical benefits of PRP in patients with knee OA [18], however, only two were double-blinded with a matched control procedure [19, 20], and neither of these evaluated the effects of PRP on objective measures of lower extremity function. This limits the ability to determine whether symptomatic benefits translate to improved mobility, which is critical given 80 % of people with OA have movement limitations and 25 % cannot perform daily activities [21].

The use of low-level light irradiation to activate peripheral blood (photo-activation) has also been shown to improve biological factors associated with osteoarthritis [22, 23]. Studies have reported that photo-activation decreases proinflammatory cytokines (interleukin 2 and 6) and increases the concentration of leucocyte-derived anti-inflammatory factors (interleukin 1 receptor antagonist) [22, 23]. As such, this activation technique could be beneficial in PRP preparations higher in leukocyte concentration. To date, only two case studies have investigated combined photo-activation and PRP (PA-PRP) in degenerative conditions, reporting symptomatic improvements in one patient with a chondral defect [24] and another with knee OA [25]. There are currently no clinical trials of PA-PRP in knee osteoarthritis. Therefore, the aim of this double-blinded, randomized, controlled pilot study was to determine the feasibility, safety and changes in pain, symptoms and lower limb functional ability following intra-articular injections of PA-PRP compared to HA in patients with mild to moderate knee OA.

## Methods

### Trial design

This was a single centre, double-blind, randomized controlled pilot study comparing PA-PRP to HA. We chose HA as the active comparator as it is one of the most commonly used injective treatments for knee OA [26], and recent network meta-analyses have demonstrated clinical benefits above intra-articular placebo injections [27, 28]. Participants were recruited from the community using online advertising and through existing databases between June and August, 2011. An information letter outlining potential risks and benefits was provided, and participants were fully informed about the testing protocol and procedures. The study was approved by the Australian Catholic University Human Research Ethics Committee, and all participants gave written informed consent prior to the commencement of testing.

### Participants

**Fig. 1:**
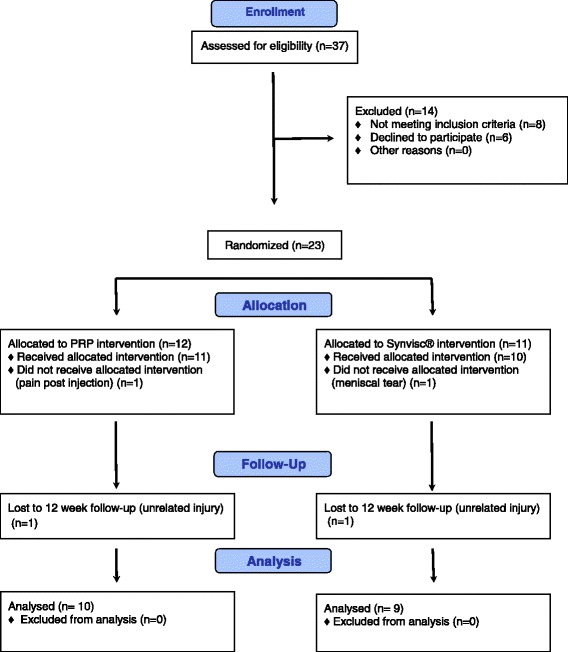
CONSORT diagram demonstrating flow of participants through the trial

Based on recommendations for determining sample sizes for pilot studies [29, 30], we enrolled 37 people with knee OA to participate in the study (Fig. 1). To be eligible, participants were required to have a diagnosis of knee OA based upon the American College of Rheumatology knee OA clinical classification criteria [31], radiographic evidence of Kellgren-Lawrence grade 2 or 3 knee OA [32] and be willing to discontinue analgesics and anti-inflammatory medications (except Paracetamol) for at least two weeks prior to commencing the intervention and for the duration of the study. Only people with mild to moderate radiographic OA disease were chosen based on recommendations from a recent systematic review of PRP in people with degenerative knee pathology [33]. Exclusion criteria were systemic or inflammatory joint disease, history of crystalline or neuropathic arthropathy, cancer or other tumour-like lesions, immunosuppression or acute infective processes, pregnancy or lactation, other intra-articular lesions or treatments in the previous six months or allergy to any test substance.

### Procedures

Group allocation and concealment was performed by an independent staff member not involved with the assessment of participants. Participants were randomly allocated following baseline data collection on a 1:1 basis using a computer generated randomization list. Both groups completed the PA-PRP collection and activation phase of the intervention as explained below, and the syringe was then provided to the independent staff member. The staff member either retained or discarded the syringe containing the PA-PRP depending on group allocation. The syringe containing the PA-PRP, or an identical looking syringe containing HA (Synvisc® Hylan G-F 20, Genzyme Biosurgery, Ridgefield, NJ, USA), was then occluded and returned to the treating doctor for administration. This process ensured the blinding of both the patient and treating doctor. All injections were performed at weekly intervals.

To obtain the PRP, 48.5 ml of the patient’s blood was collected using venipuncture, then centrifuged (Premiere XC-2000) at 2,000 rpm for five minutes. The plasma and buffy coat containing platelets was drawn from the top of the sample and placed in a sterile tube and centrifuged again at 3,000 rpm for three minutes. This double-spin approach has been shown to produce PRP that is higher in leukocytes [34], which was preferred for this study as photo-activation is thought to act at least in part by influencing the pro- and anti-inflammatory properties of leukocytes [22, 23]. Three quarters of the plasma was removed, 0.2 ml of sodium bicarbonate 8.4 % was added to the tube and the platelet pellet was reconstituted using the remaining plasma in a 3 ml syringe. To activate the PRP, the syringe containing the PRP then underwent low-level ultraviolet light irradiation (Adilight-1, Adistem Ltd.) for five minutes consistent with published protocols [23].

Participants were placed in a supine position and sterile drapes were placed around the surrounding area. Next, the participant’s symptomatic knee was cleaned with chlorhexadine and iodine solution and the knee was anaesthetised using an intra-articular injection of 5 ml of 1 % Xylocaine. Following activation, 3 ml of PA-PRP or HA was injected under ultrasound guidance (Logic i, GE Healthcare) into the symptomatic region using an anteromedial approach. Following the injection, passive flexion and extension of the knee was performed 10 times, after which the participant remained resting in the supine position for approximately 10 min. Participants were advised to take paracetamol if they experienced any pain, and to limit their weight bearing activities for the subsequent 24 h, followed by gradual resumption of normal activities.

### Outcomes

Prior to treatment, baseline demographic data was collected. Feasibility was recorded using recruitment and retention rates, and safety was assessed by recording the number and nature of adverse events. Adverse events were recorded weekly for the first month using participant phone calls, and at one and three month during the follow up assessment visits. To evaluate symptom severity, participants first completed a 100 mm Visual Analogue Scale (VAS) to rate their average knee pain over the previous week, with terminal descriptors of “no pain” and “worst pain possible” [35]. To document self-reported symptoms, participants also completed the Knee Injury and Osteoarthritis Outcome Score (KOOS) and the Knee Quality of Life 26-item questionnaire (KQoL-26). The KOOS is an OA disease-specific instrument for the assessment of patient-relevant treatment effects. The KOOS uses a five point Likert scale for scoring the three Western Ontario and McMaster Universities Osteoarthritis (WOMAC) sub-scales of Pain, Stiffness and Physical Function, with the additional inclusion of the sub-scales Quality of Life and Sport and Recreation [36]. The KQoL-26 is a self-reported quality of life instrument developed to assess the severity of knee symptoms and activity and work limitations in patients with knee injuries. It is comprised of three sub-scales, including Physical Functioning, Activity Limitations and Emotional Functioning, and uses a five point Likert scale for scoring [37].

Finally, recent research suggests that validated self-report questionnaires and objective functional tests should be employed in combination to fully assess mobility-related outcomes in people with knee OA [38]. Consequently, two objective measures of lower extremity functional ability (maximum single leg hop and number of knee bends in 30 s) were also completed by participants as described previously [38, 39]. The maximum single leg hop for distance required a hop to be performed from a starting position of balancing on one leg, and finishing position of landing and balancing on the same leg to a stationary stance position. The maximum number of knee bends performed in 30 s required the participant to balance on one leg and perform as many shallow knee bends (until they couldn’t see their toes past the bent knee, or approximately 30 degrees) as they could in a 30 s period without touching the elevated foot on the ground.

All surveys and functional tests were completed at baseline, four weeks and at a final follow-up at 12 weeks following the final treatment injection, as this timeframe has been shown to demonstrate symptomatic improvements in previous studies using other PRP preparation methods [19, 20, 40].

### Statistical analysis

The Statistical Package for Social Sciences (SPSS) software (Version 17) was used to analyse the data. Normality was assessed using skewness and kurtosis statistics and the Shapiro-Wilk test. To determine whether PA-PRP and HA had an effect on outcome variables, one way Repeated Measures Analysis of Variance (RM ANOVA) were conducted to compare baseline, four and 12 week follow-up scores for the VAS, KOOS, KQoL-26 and functional tests for each group. An analysis of covariance (ANCOVA) was used to assess between-group changes in the primary and secondary outcomes, with group allocation as the fixed factor and corresponding baseline outcome values as covariates. This technique has been reported to be the optimal method to analyse continuous data in clinical trials [41]. An alpha level of *p* < 0.05 was used to determine statistical significance.

## Results

Twenty three (62 %) of the 37 enrolled people with knee OA (males = 16, females = 7, age = 51.20 ± 12.00 years, mass = 96.35 ± 18.14 kg, height = 178.00 ± 10.10 cm, BMI = 29.24 ± 9.52) met the inclusion criteria. Two participants did not complete the allocated intervention; one from the PA-PRP group who withdrew due to minor injection-related pain and swelling which resolved without further treatment, and one from the HA group who suffered an acute meniscal tear unrelated to the treatment. Two additional participants withdrew from the study due to other unrelated injuries after the four week follow up assessments. Consequently, data was available for 21 participants (PRP = 11 participants, HA = 10 participants) for the four week follow up and for 19 participants at 12 weeks (Fig. 1). Participant characteristics are displayed in Table 1. No differences were found between the two groups (*p* > 0.05).

**Table 1:** Comparison of participant characteristics who completed the intervention by group

	PA-PRP (*n* = 11)	HA (*n* = 10)
Age, mean (SD) years	49.91 (13.72)	52.70 (10.30)
BMI, mean (SD) kg/m^2^	27.92 (11.94)	30.87 (5.64)
Previous surgery, no. (%)	5 (45 %)	8 (80 %)
Gender (Male), no. (%)	8 (72.73 %)	7 (70 %)
Symptom duration, mean (SD) years	8.50 (4.95)	15 (7.07)
Cause of osteoarthritis, no.		
Degeneration/unknown	6	4
Post joint injury/surgery	3	3
Sport related degeneration	2	3

Legend: *SD* standard deviation, *BMI* body mass index, *KL* Kellgren Lawrence, *PA-PRP* photo-activated platelet-rich plasma, *HA* hyaluronic acid

No treatment-related major adverse events were experienced by participants. Two participants from the PA-PRP group experienced minor pain and swelling during the injection period believed to be related to the injection technique. Both participants completed the injection course, and had resolution of symptoms by the following week. No further adverse events were experienced during the intervention or follow-up period.

Average pain recorded using the 100 mm VAS reduced from baseline at the four and 12 week follow up time points for both the PA-PRP and HA groups (Table 2). Although there was a slight increase in pain at the 12 weeks in the PA-PRP group, this still represented a 24 % improvement above baseline pain. Repeated Measures ANOVA revealed the reduction in pain was significant for the PA-PRP group only (*p* = 0.017). Post hoc tests showed that the reduction in pain at four weeks was significantly less than baseline (27.67 mm, 95 % CI 12.39 to 42.95, *p* = 0.003, ETA = 0.686), however the 11.4 mm reduction in VAS at 12 weeks was not statistically significant. No significant reductions in pain were found for the HA group.

**Table 2:** Mean (SD) for VAS, KOOS, KQoL and functional tests at baseline and four and 12 weeks following final injection

		PA-PRP			HA		
		Baseline (*n* = 11)	4 weeks (*n* = 11)	12 weeks (*n* = 10)	Baseline (*n* = 10)	4 weeks (*n* = 10)	12 weeks (*n* = 9)
Pain	VAS	48.09 (23.75)	19.64 (17.61)**	36.89 (25.42)	39.70 (21.90)	12.90 (14.06)	14.13 (9.30)
KOOS	Symptoms	48.70 (15.83)	57.14 (20.33)	57.86 (22.76)	62.14 (17.99)	61.07 (26.86)	80.16 (8.40)
	Pain	57.07 (11.21)	71.47 (16.67)**	68.89 (15.76)*	70.00 (11.25)	67.22 (25.55)	79.32 (9.33)
	Function	70.72 (13.64)	79.27 (15.08)	78.68 (15.87)	75.44 (12.42)	79.12 (28.63)	90.03 (7.31)**
	Sport	31.82 (20.40)	40.46 (28.32)	41.00 (27.77)	47.00 (28.69)	46.50 (33.75)	64.44 (23.64)
	QoL	30.11 (18.92)	40.89 (27.55)	38.75 (28.38)	41.87 (13.51)	42.50 (21.21)	54.86 (9.77)
KQoL	Physical	57.72 (18.35)	65.00 (18.14)*	68.83 (18.64)**	71.16 (14.91)	68.33 (27.54)	80.55 (13.46)
	Activity	59.09 (23.33)	72.73 (16.79)	70.00 (22.23)	75.50 (15.71)	78.50 (29.16)	88.89 (7.41)
	Emotional	46.97 (26.69)	58.71 (23.68)*	58.75 (29.49)**	58.75 (24.25)	67.08 (29.88)	75.00 (16.00)
Functional tests	Hops	46.64 (33.04)	57.64 (41.36)*	79.33 (34.17)**	55.50 (35.43)	51.50 (39.49)	79.25 (38.04)
	Knee bends	19.45 (8.25)	22.27 (8.37)	31.44 (7.96)**	20.50 (13.23)	25.30 (16.60)	31.13 (15.63)

Legend: *VAS* visual analogue score, *KOOS* knee osteoarthritis outcome score, *QoL* quality of life, *KQoL* knee quality of life, *PA-PRP* photo-activated platelet-rich plasma, *HA* hyaluronic acid

*Indicates statistically significant (*p* < 0.05) improvement from baseline

**Indicates statistically significant (*p* < 0.01) improvement from baseline

For the PA-PRP group, significant improvements were found at both follow up time points in the KOOS Pain subscale (4 weeks: 13.33, 95 % CI 4.66 to 22.01, *p* = 0.007; 12 weeks: 11.67, 95 % CI 2.99 to 20.34, *p* = 0.014, ETA = 0.624), the KQoL-26 Physical subscale (4 weeks: 8.50, 95 % CI = 1.84 to 15.16, *p* = 0.018; 12 weeks: 9.33, 95 % CI 4.56 to 14.11, *p* = 0.002, ETA = 0.706) and the KQoL Emotional subscale (4 weeks: 12.08, 95 % CI 3.26 to 20.91, *p* = 0.013; 12 weeks: 9.58, 95 % CI 17.11 %, *p* = 0.009, ETA = 0.715) (Table 2). For the HA group, significant improvements were only found in the KOOS Function subscale at 12 weeks (14.05, 95 % CI 18.59 to 24.15, *p* = 0.008, ETA = 0.602). Table 2 also demonstrates that the PA-PRP group significantly improved both hopping (4 weeks: 13.44, 95 % CI = 2.23 to 24.66, *p* = 0.025; 12 weeks: 22.33, 95 % CI 11.86 to 32.80, *p =* 0.001, ETA = 0.799) and knee bend (12 weeks: 9.78, 95 % CI 5.00 to 14.56, *p =* 0.002, ETA = 0.756) performance, whereas changes in physical function in the HA group were not found to be significant. To investigate between-group differences in mean change scores on the 100 mm VAS, KOOS, KQoL-26 and functional tests, ANCOVA was performed adjusting for baseline scores (Table 3). No significant between-group differences were found for any of the self-reported measures or for either of the functional tests (*p >* 0.05).

**Table 3:** Adjusted mean difference (95% confidence intervals; CI) between the two groups for VAS, KOOS, KQoL and functional tests at baseline and four and 12 weeks following final injection

		4 weeks		12 weeks	
		Mean difference (95 % CI)	*P* value	Mean difference (95 % CI)	*P* value
Pain	VAS	1.90 (-13..80 to 17.60)	0.79	18.81 (-0.62 to 38.24)	0.06
KOOS	Symptoms	7.52 (-17.20 to 32.23)	0.53	−15.57 (-34.12 to 2.99)	0.09
	Pain	15.64 (-9.81 to 41.10)	0.21	−4.30 (-19.76 to 11.16	0.56
	Function	4.44 (-17.00 to 25.88)	0.67	−8.93 (-19.92 to 2.07)	0.10
	Sport	0.66 (-29.31 to 30.62)	0.96	−13.68 (-36.38 to 9.02)	0.22
	QoL	11.17 (-6.22 to 28.57)	0.19	−8.09 (-25.11 to 8.93)	0.33
KQoL	Physical	10.90 (-9.11 to 30.91)	`0.27	−0.41 (-8.59 to 7.77)	0.92
	Activity	6.46 (-16.09 to 29.01)	0.55	−11.01 (-25.59 to 3.57)	0.13
	Emotional	0.18 (-19.76 to 20.11)	0.99	−11.60 (-28.49 to 5.28)	0.17
Functional tests	Hops	17.23 (-19.26 to 53.72)	0.33	8.93 (-5.26 to 23.13)	0.20
	Knee bends	−0.81 (-9.29 to 7.68)	0.84	1.16 (-9.11 to 11.43)	0.81

Legend: *VAS* visual analogue scale, *KOOS* knee osteoarthritis outcome score, *QoL* quality of life, *KQoL* knee quality of life, *CI* confidence intervals

## Discussion

This was a double-blind randomized controlled pilot study comparing intra-articular injections of a novel form of PRP activated with ultraviolet light compared to HA. The results demonstrate the feasibility of this technique in people with knee OA and show no serious adverse events were reported. There was a significant decrease in VAS pain for the PA-PRP group at four weeks that was greater than the minimal clinically important improvement level of 19.9 mm. The 24 % decrease below baseline pain levels at 12 weeks was not found to be statistically significant and no improvements in pain were found in the HA group. The PA-PRP group also demonstrated significant improvements in the KOOS Pain subscale at four and 12 weeks, and significant improvements in the KQoL-26 Physical and Emotional subscales. In contrast, the only significant improvement in the HA group was in the KOOS Functional subscale at 12 weeks. Finally, whilst the PA-PRP group significantly improved their performance on the functional measures of hopping and knee bend at 12 weeks, no significant improvements in lower extremity function were found in the HA group. Despite these improvements in the PA-PRP compared to the HA group, there were no significant between-group differences on any measures after adjusting for baseline variation. These preliminary results provide initial feasibility, safety and treatment data that may be used to inform a future large clinical trial to investigate whether this novel form of photo-activated PRP has symptomatic and functional benefits in people with knee OA.

Of the 23 people who met the inclusion criteria, two participants did not complete the allocated intervention and two additional participants withdrew from the study following the four week follow-up appointment. These four patients were evenly spread between groups, and with the exception of one, were all due to new injuries unrelated to the treatment. One of the PA-PRP patients however did withdraw due to minor injection-related soreness, whilst another two also reported minor pain and swelling following an injection. In all instances, symptoms resolved within a week and no further adverse events were reported during the intervention or follow-up period.

Previous RCTs have investigated the efficacy of PRP compared to HA or saline [19, 20, 34, 40, 42, 43] however to our knowledge this is the first to objectively evaluate lower limb function and the only one to use photo-activation of PRP. Photo-activation of peripheral blood has been previously shown to improve the inflammatory cytokine profile of healthy adults [23] and people with psoriasis [22], and therefore has the potential to improve outcomes in OA where pro-inflammatory cytokines are critical mediators in the pathophysiology of the disease [44]. Combined with PRP, which releases growth factors and other proteins responsible for tissue repair and inflammatory modulation [45, 46], this may offer a novel method for improving symptoms and function in people with knee OA. Our results provide mixed support for this new treatment approach. Although the PA-PRP group showed significantly greater improvement in self-reported symptoms and lower limb physical function compared to HA, between-group differences were not significant. It is possible that the small sample size of our pilot study may have reduced statistical power making definitive between-group conclusions difficult.

The lack of between-group differences found in our pilot study may also be partly due to our PRP preparation method. We used a double spin technique which has previously been shown to produce PRP that is higher in leukocyte concentration when compared to PRP prepared using a single spin technique [34]. Indeed, our findings are consistent with two recent RCTs reporting comparable between-group improvements in self-reported pain and symptoms following injections of either PRP prepared using a leukocyte-rich technique or HA [19, 26]. In contrast, two recent studies investigating the effects of PRP prepared using a leukocyte-poor technique, and a recent meta-analysis [47], have suggested this form of PRP produces improvements in self-reported symptoms above that of HA. Cerza and colleagues [40] showed significantly improved overall WOMAC score at 12 and 24 weeks in knee OA patients who had injections of PRP prepared using a leukocyte-poor technique compared to patients who received HA. Similarly, Sanchez and colleagues [43] reported that patients with knee OA who received leukocyte-poor PRP had significant benefits at 24 weeks compared to those who received HA. The findings of our study, and those from these previous clinical trials, suggests that leukocyte concentration of PRP may play a crucial role in clinical outcomes achieved for knee OA patients. However, future studies should explore the effects of photo-activation in both leukocyte-rich and leukocyte-poor PRP preparations given photo-activation is thought to act at least in part by modulating the pro- and anti-inflammatory properties of leukocytes [22, 23].

There are some limitations that may have influenced the outcomes of our study. Firstly, as mentioned the small sample size of this pilot trial reduces statistical power which may make more definitive conclusions problematic, and the relatively short follow-up period of our study may have been too brief to determine between-group differences. A larger clinical trial is needed to confirm our results. Furthermore, we did not include a minimum VAS pain score as part of our screening criteria, resulting in greater variation in baseline pain and self-reported symptoms. This produced high baseline standard deviations which would have decreased the probability of finding differences between the PRP and HA groups, particularly with the small sample size. Finally, we did not examine how photo-activation modifies PRP. Given the benefits of photo-activation have been attributed to its influence upon the concentration of proinflammatory cytokines and leucocyte-derived anti-inflammatory factors [22, 23], and the potential role of leukocyte concentration in PRP, this warrant further investigation.

## Conclusions

This randomized controlled pilot study provides feasibility and safety data for the use of PA-PRP in people with knee OA that may help to inform a larger clinical trial. Although under-powered to assess efficacy, our preliminary results also provide some evidence that PA-PRP improves self-reported pain, subscales of the KOOS and KQoL-26, and tests of lower extremity functional ability in knee OA patients. However these improvements were not found to be significantly greater than those of the HA group. Future clinical trials with larger sample sizes and longer follow up periods should investigate whether this novel photo-activated PRP method improves symptoms and function over that of HA in people with knee OA.

## Abbreviations

ANCOVA: analysis of covariance
CI: confidence interval
HA: hyaluronic acid
KOOS: Knee Osteoarthritis Outcome Score
KQoL: Knee Quality of Life
PA-PRP: photo-activated platelet-rich plasma
PRP: platelet-rich plasma
RCT: randomized controlled trial
RM ANOVA: Repeated Measures Analysis of Variance
VAS: visual analogue pain
WOMAC: Western Ontario and McMaster Universities Osteoarthritis
